# Identification of two novel mutations in *CDHR1* in consanguineous Spanish families with autosomal recessive retinal dystrophy

**DOI:** 10.1038/srep13902

**Published:** 2015-09-09

**Authors:** Konstantinos Nikopoulos, Almudena Avila-Fernandez, Marta Corton, Maria Isabel Lopez-Molina, Raquel Perez-Carro, Lara Bontadelli, Silvio Alessandro Di Gioia, Olga Zurita, Blanca Garcia-Sandoval, Carlo Rivolta, Carmen Ayuso

**Affiliations:** 1 Department of Medical Genetics, University of Lausanne, Lausanne, Switzerland; 2 Department of Genetics, Instituto de Investigacion Sanitaria-University Hospital Fundacion Jimenez Diaz (IIS – FJD, UAM), Madrid, Spain; 3 Centro de Investigacion Biomedica en Red (CIBER) de Enfermedades Raras ISCIII, Madrid, Spain; 4 Department of Ophthalmology, University Hospital Fundacion Jimenez Diaz, Madrid, Spain

## Abstract

Inherited retinal dystrophies present extensive phenotypic and genetic heterogeneity, posing a challenge for patients’ molecular and clinical diagnoses. In this study, we wanted to clinically characterize and investigate the molecular etiology of an atypical form of autosomal recessive retinal dystrophy in two consanguineous Spanish families. Affected members of the respective families exhibited an array of clinical features including reduced visual acuity, photophobia, defective color vision, reduced or absent ERG responses, macular atrophy and pigmentary deposits in the peripheral retina. Genetic investigation included autozygosity mapping coupled with exome sequencing in the first family, whereas autozygome-guided candidate gene screening was performed by means of Sanger DNA sequencing in the second family. Our approach revealed nucleotide changes in *CDHR1*; a homozygous missense variant (c.1720C > G, p.P574A) and a homozygous single base transition (c.1485 + 2T > C) affecting the canonical 5’ splice site of intron 13, respectively. Both changes co-segregated with the disease and were absent among cohorts of unrelated control individuals. To date, only five mutations in *CDHR1* have been identified, all resulting in premature stop codons leading to mRNA nonsense mediated decay. Our work reports two previously unidentified homozygous mutations in *CDHR1* further expanding the mutational spectrum of this gene.

Inherited retinal dystrophies (IRDs) encompass a large group of progressive neurodegenerative diseases with functional consequences on retinal physiology that lead eventually to significant visual handicap. IRDs can be broadly characterized on the basis of the sequential order and relative severity of the dysfunction or loss of either photoreceptors, and include cone dystrophies (CD), cone–rod dystrophies (CRDs) and retinitis pigmentosa (RP)^1^. When primary cone dysfunction (CD/CRDs) is present, initial symptoms include early and progressive loss of central visual acuity, defective color vision and photophobia, whereas RP causes in patients night blindness as the first noticeable symptom^2^,^3^,^4^. Genetically, IRDs display all Mendelian inheritance patterns, i.e. autosomal recessive (ar), autosomal dominant (ad), or X-linked (Xl). To date, mutations in more than 105 genes (RetNet; http://www.sph.uth.tmc.edu/retnet/) have been identified to cause several forms of inherited retinal dystrophies. IRDs are also characterized by genetic and clinical overlap, for which for instance mutations in specific genes can result in different diseases^4^,^5^.

Functionally, a significant proportion of genes associated with IRDs encode proteins with key roles in the maintenance of photoreceptor structure and integrity. One of them is the cadherin-related family member 1 (*CDHR1*, OMIM *609502, formerly known as *PCDH21*), a member of the calcium-dependent cadherin superfamily of homophillic cell-adhesion proteins. Structurally, these molecules are integral proteins, primarily characterized by the presence of multiple (up to 34) cadherin repeats, i.e. large extracellular calcium (EC) binding domains that determine individual cadherin’s functional profile^6^,^7^. Cadherins are evolutionary conserved, and even subtle aminoacid changes have been found to impact their adhesive binding specificity with protein partners. Of note, mutations in four cadherin family members (among which *CDHR1*) have been identified to cause retinal dystrophy^8^,^9^,^10^,^11^.

CDHR1 is composed of six cadherin repeats, one transmembrane, and one intracellular domain. The protein is present in a small fraction of neuronal tissues, including the olfactory bulb and the retina^12^,^13^. Within the retina, CDHR1 appears to exert its function at the base of the photoreceptor’s outer segment, and especially at the junction between the inner- and outer segments, opposite to the connecting cilium^14^,^15^. A *cdhr1*^-/-^ mouse model exists, for which disruption of the gene leads to compromised structures of cone and rod outer segments and progressive photoreceptor degeneration^15^, making *CDHR1* a prime candidate disease gene for IRDs in humans.

Historically, Bolz and colleagues^16^ proceeded first with a comprehensive screening of the *CDHR1* gene in a large cohort of IRD patients and identified two missense variants with inconclusive pathogenic potential, since they were heterozygous with no second pathogenic allele in either of the carrier persons. To date, further studies have revealed only a handful of cases with mutations in *CDHR1* causing either arCRD or a clinically related form of retinopathy, sometimes referred to as “CDHR1-related retinopathy” affecting primarily but not solely the cone photoreceptors^2^,^11^,^17^,^18^,^19^. The majority of *CDHR1* mutations, ascertained from populations with different ethnic background, likely result in nonsense mediated mRNA decay and thus in reduced or in no protein content in affected individuals. In this work, we describe two consanguineous Spanish families that were investigated by combining autozygosity mapping with candidate gene analysis, either by using exome sequencing or “classical” sequencing analysis by the Sanger method. Patients also underwent detailed clinical evaluation by using retinal imaging and electroretinography techniques. Finally, our study yielded two previously-undescribed homozygous mutations in *CDHR1*.

## Methods

### Patients

**Figure 1:**
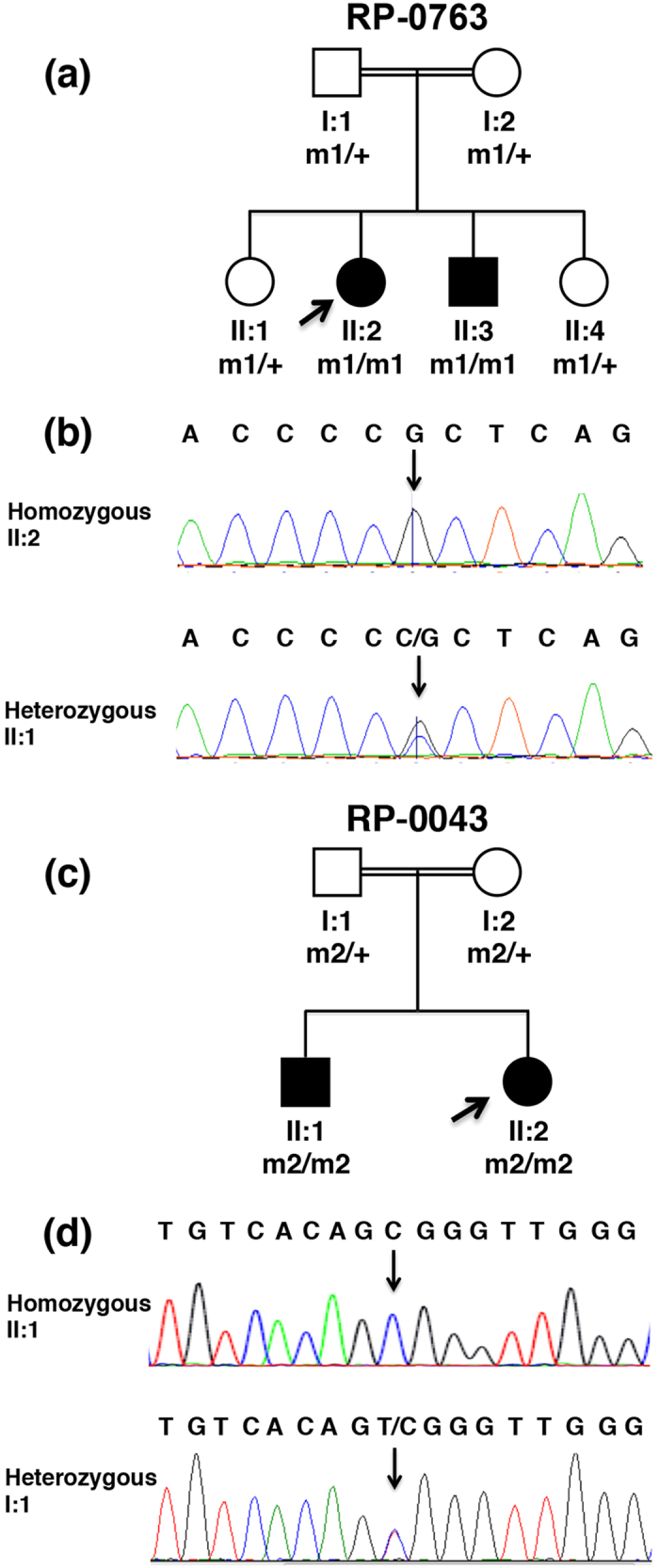
Pedigrees of patients analyzed and mutations identified in this work. **(a)** Pedigree of family RP-0763. The parents are first cousins. Open and closed symbols represent unaffected and affected individuals, respectively. m1/m1 refers to the homozygous presence of the mutation c.1720C>G in *CDHR1* (NM_033100.3), whereas m1/+ refers to its heterozygous presence. The arrow indicates the proband for this family. **(b)** Chromatograms of Sanger DNA sequencing surrounding the *CDHR1* variant c.1720C>G are shown for patient II:2 and the healthy carrier individual II:1. **(c)** Pedigree of family RP-0043 in which parents are also first cousins. m2/m2 refers to the homozygous presence of the mutation c.1485 + 2T>C in *CDHR1* (NM_033100.3), whereas m2/+ refers to its heterozygous presence. The arrow indicates the proband for this family. **(d)** Chromatogram of Sanger DNA sequencing surrounding the *CDHR1* variant c.1485 + 2T >C is shown for patient II:1 and the healthy parent I:1.

Six members of family RP-0763 and four members of RP-0043, both from Spain, were ascertained for this study (Fig. 1). As controls, 165 healthy and unrelated subjects from Spain were included.

This study was carried out in accordance with the tenets of the Declaration of Helsinki and was approved by the Institutional Review Boards of the University of Lausanne, Switzerland and the Clinical Research Ethics Committee of the Fundacion Jimenez Diaz University Hospital in Spain. Written informed consent was obtained from the subjects who participated in this study and donated their blood for research. Each individual was anonymized by assigning to them a numeric ID; confidentiality and protection of data were ensured by applying international recommendations and current Spanish legislation (Ley de Investigacion Biomedica 14/2007 and LOPD).

### Clinical examination

Affected individuals underwent a full ophthalmic examination including evaluation of best-corrected visual acuity (BCVA), intraocular pressure, ocular motility, pupillary reaction, biomicroscopic slit-lamp examination, and dilated fundus examination. The color vision was examined by the 28 HUE Farnsworth or Ishihara tests. Visual function was evaluated by static perimetry, optical coherence tomography (Cirrus, Carl Zeiss Meditec Inc., Dublin, CA) and Ganzfeld electroretinography, according to the guidelines of the International Society for Clinical Electrophysiology of Vision.

### Genetic analysis

Genomic DNA was extracted from 1 ml of whole blood using an automated DNA extractor (Magna Pure Compact, Roche, Basel, Switzerland) following the manufacturer’s instructions.

Whole genome homozygosity mapping was performed using high-resolution commercial SNP arrays from Affymetrix (Affymetrix, San Diego, CA, USA) (Genome Wide Human SNP array) and Illumina (Illumina, Santa Clara, CA, USA) (Omni Whole Genome arrays HumanCytoSNP-12) for RP-0763 and RP-0043, respectively. Arrays were processed according to the manufacturer’s protocols. Genome-wide autozygosity mapping was performed using the Linkage Disequilibrium - Hidden Markov Model algorithm (LD-HMM)^20^ through the dCHIP software^21^.

For mutation analysis, specific primers were used to PCR-amplify the entire *CDHR1* open reading frame (ORF), consisting of 17 exons and of their exon-intron boundaries. Primer sequences were described previously^11^,^16^, except for exon 15, for which a distinct primer pair was designed (forward: 5′-ACACCCATGCCTATGTGCTC-3′, reverse: 5′-TATCTCTTGGAGCTGCTGGA-3′). PCR amplification proceeded under standard conditions.

Mutation screening was performed by direct Sanger sequencing with the Big Dye terminator cycle sequencing kit on an ABI 3130xl Genetic Analyzer (PE Applied Biosystems, Foster City, CA, USA). The DNA control samples for the c.1720C >G mutation were screened with a restriction endonuclease-based assay with *Aci*I (New England Biolabs, Beverly, MA, USA).

In addition, DNA control samples for c.1485 + 2T >C in exon 13 were screened by High Resolution Melting (HRM) analysis according to an optimized protocol. Real-time PCR and HRM were consecutively done on a LightCycler 480 Real-Time PCR System (Roche, Basel, Switzerland) in one single run, and all reactions were performed in duplicate. PCR and HRM conditions are available on request. PCR products displaying abnormal HRM profiles were further analysed by direct Sanger sequencing.

Exome sequencing was performed in individual II:2 from family RP-0763 (Fig. 1). Exome capture and library construction were performed using the Agilent SureSelect Human All Exon v4 Kit (Agilent, Wokingham, UK) using 6 μg of genomic DNA. Libraries were sequenced on an Illumina HiSeq 2000 (Illumina, San Diego, CA) generating 100 bp paired-end reads. Reads were aligned to the hg19 human reference sequencing using Novoalign (Novocraft, Selangor, Malaysia) version 2.05. The Genome Analysis Toolkit (GATK)^22^ was used for refining mapping around small insertions/deletions, base quality score recalibration and variant calling. In order to detect the potential disease-causing variant(s), a proprietary filtering pipeline was implemented based on homemade Perl scripts as previously described^23^. Finally, variants were prioritized on the basis of their presence in shared regions of autozygosity, taking into assumption the inheritance of identical genotypes from a single founder from both related patients.

### *In silico* assessment of mutation pathogenicity

The potential consequences of the mutation c.1485 + 2T >C on the normal splicing of its neighboring exons were analyzed *in silico* using the NetGene 2 Server^24^ and Human Splicing Finder^25^. In addition Polymorphism Phenotyping v2 (Polyphen-2)^26^, Sorting Intolerant from Tolerant (SIFT)^27^ and Mutation Taster^28^ were used in order to evaluate the putative pathological nature of the missense variant that we report in this study.

### Amino acid conservation

CDHR1 protein sequences from different species including human (*H. sapiens*, NP_149091.1), mouse (*M. musculus*, NP_570948.1), cow (*B. taurus*, NP_777248.1), chicken (*G. gallus*, NP_001001759.1), Xenopus (*X. tropilcalis*, XP_002933948.2) and zebrafish (*D. rerio*, NP_001005402.1) were aligned using the CLC Genomics Workbench (CLCbio, Qiagen, Boston, USA) in order to check the evolutionary conservation of their substituted amino acid residues.

## Results

### Clinical examination

**Figure 2:**
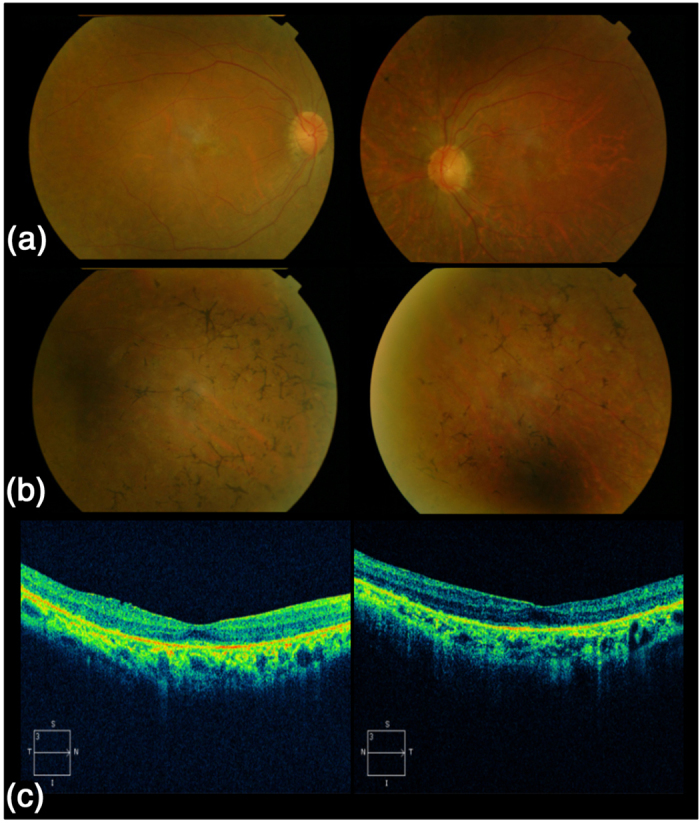
Fundus photographs and optical coherence tomography (OCT) of affected individual II:2 from family RP-0763. **(a)** Fundus photograph taken at the age of 45 (left and right eye) demonstrating a pale optic disc and attenuated blood retinal vessels. Macular involvement is noted with the presence of spotted hyperpigmentation (yellowish dots) and annular RPE with central sparing of the fovea (bull’s eye maculopathy). **(b)** Fundus photograph of the retinal periphery (left and right eye) demonstrating the presence of pigmentary changes in the form of scarce bone spicule pigmentation. **(c)** OCT of left and right eye demonstrating a decreased foveal depression.

**Figure 3:**
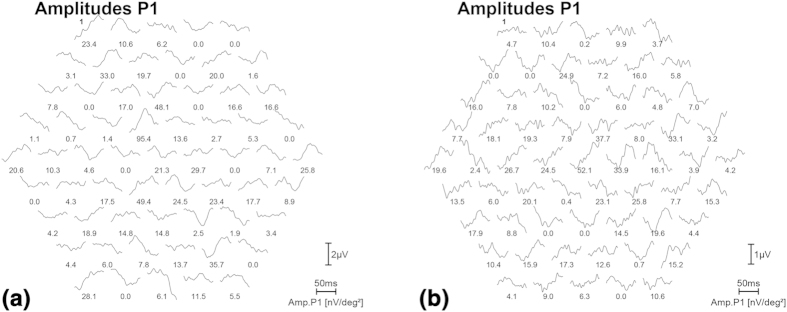
Multifocal electroretinography (mfERG) of patient II:2 from family RP-0763, for the left (a) and right (b) eye. The mfERG shows a reduced amplitude in all records at the age 45.

For the index case (II:2) of family RP-0763 (Fig. 1) the initial diagnosis was made at the age of 34. Photophobia, photopsia and color vision disturbances were the first noticeable symptoms, followed by night blindness and peripheral visual field loss. At the time of the last ophthalmologic examination (45 years), the best-corrected visual acuity was 0.8 for the right eye (OD) and 0.7 for the left eye (OS). Posterior subcapsular cataract was found. The 28 HUE Farnsworth test showed nonspecific mild abnormalities. She presented with a tubular visual field with a small island of central vision accompanied by temporal island at the age of 42 being reduced to absolute scotoma at the age of 45 (Supplementary Figure S1). The fundus showed a pale disc, narrowed vessels, scarce bone spicule pigmentation in the mid-periphery, yellowish dots in macula and macular retinal pigment epithelium degeneration (Fig. 2). Full-field electroretinogram was nonrecordable and for the multifocal (mf) electroretinography the amplitude was decreased in all records (Fig. 3). The OCT showed bilateral thinning of the fovea (Fig. 2). Clinical data are summarized in Table 1. The affected sibling (II:3) was not available for the ophthalmic examination.

The initial diagnosis of the index case (II:1) of family RP-0043 (Fig. 1) was made in her third decade of life. Night blindness and field constriction were the first noticeable symptoms. At 33 years of age, visual field was symmetrically reduced to 10 degrees and the visual acuity was 0.2 OD and 1 OS. The patient was amblyopic since childhood. Fundus examination showed pale discs, narrow vessels, bone spicule pigmentation in the mid-periphery and a bull’s eye macular phenotype resulting in annular retinal pigment epithelium (RPE) atrophy with central sparing of the fovea. Full-field electroretinogram was non-recordable and in multifocal electroretinography (mfERG) the amplitude was diminished in all instances. After further ophthalmologic examination (at age 49 years) the patient showed light perception visual acuity for both eyes (Table 1).

For the affected sibling (II:2), the initial diagnosis was made at the age of 32. Visual field presented central scotoma and the visual acuity was 0.12 OD and 1 OS. The refractive error was −3 sphere, −1.50 cylinder OD and −2 sphere OS. The 28 HUE Farnsworth test showed alterations in color vision. The fundus showed small optic papillae, unstructured macula and atrophy in the periphery (Table 1).

### Genetic analysis

Both families reported in this study are consanguineous and retinal dystrophy segregates in an autosomal recessive mode. Based on this information, we performed genome-wide, SNP-based autozygosity mapping. Our analysis revealed several large autozygous regions that were shared among the affected siblings in the two families (Table 2). In particular, regions containing more than 300 consecutive homozygous SNPs, on average corresponding to a genomic size of 1 Mb or larger, were prioritized.

In family RP-0763, autozygosity analysis resulted in five large autozygous regions (Table 2). The third largest autozygous interval, which encompasses a genomic region of 5.7 Mb on chromosome 10 (hg19: 85.7-91. Mb) contains *CDHR1*. Following the evaluation of the exome sequencing data of patient II:2 (family RP-0763) we identified a homozygous mutation c.1720C >G (p.P574A) in this gene.

**Figure 4:**
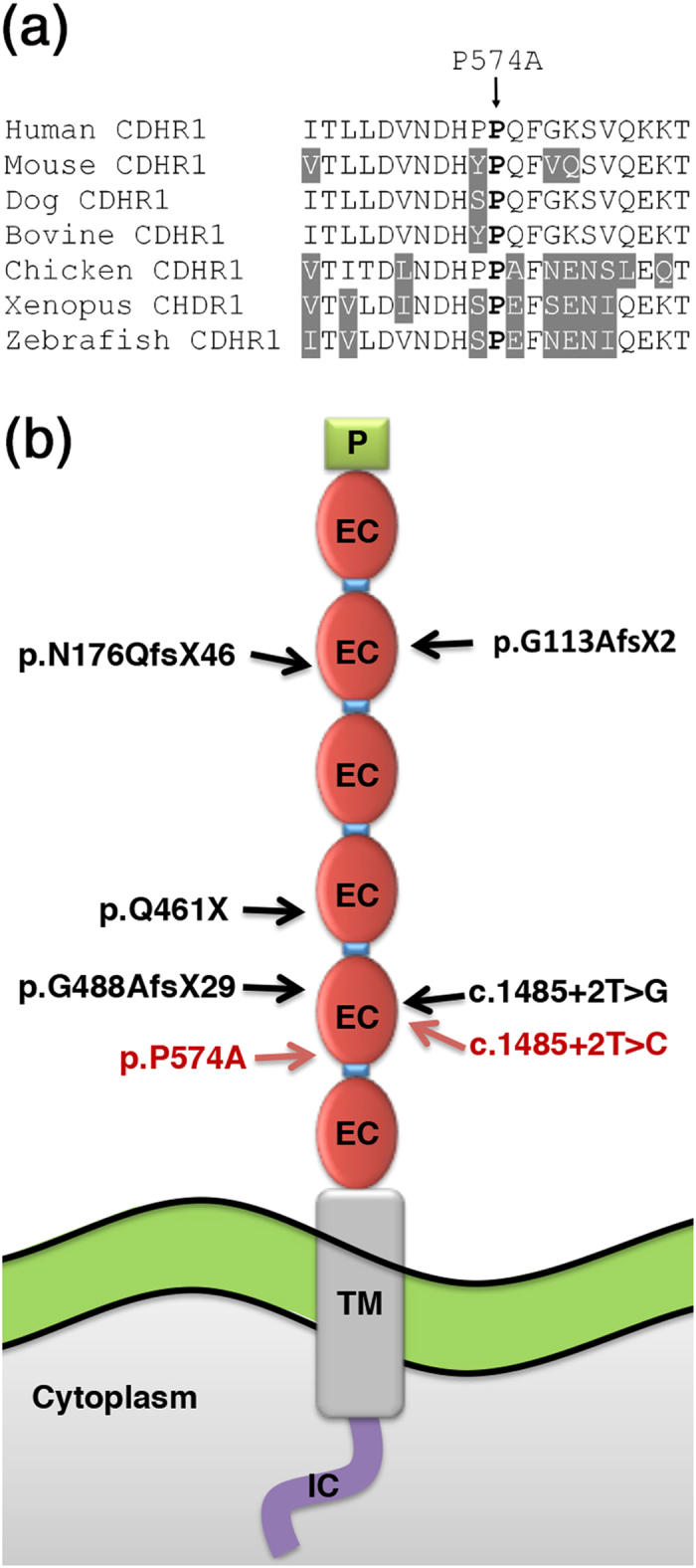
Amino acid sequence alignment of human CDHR1 in the area spanning the missense variant p.P574A and topology of the CDHR1 protein indicating the position of all reported mutations. (**a**) Amino acid sequence alignment of human CDHR1 with orthologous proteins from mouse, dog, cow, chicken, frog, and zebrafish. Ten upstream and 10 downstream amino acids from the missense variant P574A are depicted. Residues identical to the human sequence across all sequences are black on a white background whereas different amino acids are indicated in white on a grey background. The amino acid residue at the position of the missense change is indicated in bold. (**b**) Topology of the CDHR1 protein and the position all reported mutations. The intracellular domain (IC) is shown as a purple line; the transmembrane domain (TM) is indicated in a box with grey background. The ectodomains (EC) are indicated in red oval shapes with the linker regions as blue boxes in between the ectodomains. Mutations reported in previous studies are indicated depicted in black whereas mutations identified in this work are indicated in red.

Dideoxy DNA sequencing confirmed the presence of this variant which co-segregated with retinal dystrophy in the pedigree (Fig. 1). The mutation was not detected in 165 healthy control individuals, in an internal control cohort containing whole exome and/or whole genome sequencing data from 350 unrelated individuals or in any other public database, including the one from 1000 Genomes Project^29^, the Exome Variant Server (EVS, http://evs.gs.washington.edu/EVS) and the Exome Aggregation Consortium (ExAC, http://exac.broadinstitute.org), which contains sequencing data from more than 61,000 unrelated individuals. The homozygous p.P574A alteration affects a proline residue, fully conserved from fish to human (Fig. 4a), and is located in the fifth cadherin repeat (Fig. 4b). Furthermore, c.1720C is an evolutionary highly conserved nucleotide with a phyloP score of 5.65 (threshold for significance >0.95). Therefore, the substitution of an alanine for this proline residue is likely to affect the CDHR1 function. Furthermore, the *in silico* analysis of p.P574A using three different online tools predicted this mutation to be probably damaging by Polyphen (score 0.998), deleterious by SIFT (score 0.00) and disease causing by Mutation Taster (score 0.999).

In family RP-0043, autozygosity analysis resulted in seven large autozygous regions (Table 2). The largest autozygous interval, which encompasses a genomic region of 47 Mb (hg: 72-118.9 Mb) contains *CDHR1*. Several additional genes known to be involved in inherited retinal dystrophies were also within these intervals, namely *LRAT* underlying Leber Congenital Amaurosis (LCA)^30^ and *AILP1* underlying LCA, juvenile RP and autosomal dominant CRD^31^,^32^. However, since the phenotypic picture of both affected siblings is consistent with autosomal recessive retinal degeneration resembling the phenotypic spectrum elicited by *CDHR1* mutations, we considered *CDHR1* as a prime disease gene candidate to be screened in this family.

Indeed, sequence analysis by the Sanger method of the gene’s ORF and intron-exon boundaries, initially in patient II:2, identified the homozygous nucleotide change c.1485 + 2T >C, which affects one of the two completely conserved nucleotides of the canonical 5' splice site of intron 13 and therefore is likely to have a major effect on *CDHR1* splicing. The mutation was also present homozygously in the affected sibling and in heterozygous state in the healthy parents (Fig. 1). Similarly to c.1720C >G, the mutation was neither detected in control individuals, nor it has been reported present in the 1000 Genomes Database or any other public database. It is of note that the exact same nucleotide position has been previously reported to be mutated in an Israeli Christian Arab family although the base substitution was different (c.1485 + 2T >G)^17^. *In silico* assessment of c.1485 + 2T >C using two distinct web-based platforms (NetGene 2 Server and Human Splicing Finder) predicts the practical abolishment of the donor splicing site (Table 3).

## Discussion

In this study we identified two consanguineous Spanish families segregating autosomal recessive retinal degeneration and harboring two previously unidentified mutations in *CDHR1*. In particular, genetic analysis in family RP-0763 revealed a homozygous, and the sole missense mutation in exon 15 (c.1720C >G, p.P574A) reported so far in this gene being associated with any type of ocular pathology. In family RP-0043 we report a homozygous splice site mutation c.1485 + 2T >C, having an impact on the second fully conserved nucleotide position of the donor splice site of exon 13.

Clinically, the diagnosis of these patients is consistent with a form of CDHR1-related retinopathy. Different autosomal recessive phenotypes have been associated with mutations in the *CDHR1* gene, ranging from RP to CRD^2^,^11^,^17^,^18^,^19^. For the RP-0763 family, although the index case (II:2) refers photophobia, photopsia and color vision disturbances as first noticeable symptoms, she keeps a good visual acuity. Therefore, the phenotype of this patient seems to resemble more RP rather than CRD, or a diffuse retinal dystrophy involving at the same time cones and rods. Overall, the phenotype of family RP-0763, harboring the missense mutation p.P574A, is less severe, at least in terms of BCVA, when compared with RP-0043 and the phenotypes described in literature^2^,^11^,^17^,^18^,^19^.

CDHR1 has been shown to be an important protein not only for photoreceptor homeostasis but also for photoreceptor development. It is present at the base of the backbone of the photoreceptor outer segment, localized at the developing discs. Its precise function has not been fully elucidated, but it has been shown that the role of CDHR1 is orchestrated via the use of its extracellular cadherin domains^33^,^14^. Ablation of *cdhr1* in a mouse model results in functional consequences for the photoreceptors, leading to disarray of the outer segment and consequent photoreceptor degeneration and death^15^. To date, seven different mutations (including the ones reported in this study) have been identified in *CDHR1* (Fig. 4b)^2^,^11^,^17^,^18^,^19^. Interestingly, six of them are reported to result in premature stop codons which most likely lead to mRNA nonsense mediated decay and thus to no or trace protein product.

In CDHR1, the p.P574A mutation is topologically located at the end of the fifth ectodomain (EC), overlapping the linker region that joins the two neighboring cadherin ECs. Proline 574 is highly conserved across species (Fig. 4a), an element that denotes its functional importance over evolution at this specific location. The linker regions in cadherins are hypothesized to play a crucial role in protein stability but also in their structural integrity and function. They maintain the local ectodomain architecture via interactions with Ca^2+^, reinforcing the resulting EC suprastructures and preventing protein instability and immature proteolysis^34^,^35^. The replacement of proline with alanine, two residues with completely different biochemical profiles can be assumed to be destructive for the different functions that the linker region exerts. Moreover, corroborating the notion of the pathogenic nature of this mutation, three different independent *in silico* web-based tools (SIFT, Polyphen and Mutation Taster) predict with high score the p.P574A missense change as disease causing.

The c.1485 + 2T >C is the second splice site mutation reported in *CDHR1* to date^17^, affecting the same nucleotide position at the boundaries of exon 13 and intron 13 as previously reported. However, the base change differs. Based on the *in silico* prediction, the canonical splicing site is significantly knocked down by this DNA change, likely leading to incorrect splicing and to an aberrant CDHR1 mRNA that should be subject to mRNA nonsense mediated decay.

In summary, in this work we delineate the retinal pathology of two families segregating autosomal recessive retinal dystrophy due to two previously undescribed mutations in *CDHR1*, one of which is the first pathogenic missense change described to date in this gene. With our findings, we provide further insight into the disease clinical and molecular portrait attributed to *CDHR1*.

## Additional Information

**How to cite this article**: Nikopoulos, K. *et al*. Identification of two novel mutations in *CDHR1* in consanguineous Spanish families with autosomal recessive retinal dystrophy. *Sci. Rep*. **5**, 13902; doi: 10.1038/srep13902 (2015).

## Supplementary Material

Supplementary Information

## Figures and Tables

**Table 1:** Clinical features of patients with mutations in *CDHR1*.

**Family Patient ID**	**Genotype**	**First symptoms and course**	**BCVA OD/OS**	**Visual Field OD/OS**	**ERG**	**Fundus aspect**	**28 HUE Farnsworth or Ishihara test**	**OCT**	**Additional findings**
RP-0763: II:2	p.P574A/ p.P574A	Photophobia, photopsia and color disturbances (30 yrs), Progressive loss of VA (33 yrs), NB (33 yrs) and field constriction (34 yrs)	0.8/0.7	Tubular visual field with a small island of central vision and a temporal island (42 yrs). Absolute scotoma (45 yrs)	Full field was non-recordable and mfERG amplitude was decreased in all records.	Pale optic disc, narrow vessels, scarce bone spicule pigmentation in mid-periphery, yellowish dots in macula and bull’s eye maculopathy	Non-specific mild abnormalities	Decreased foveal depression	Posterior subcapsular cataract
RP-0043: II:1	c.1485 + 2T>C/ c.1485 + 2T>C	NB (26 yrs) and field constriction (26 yrs)	0.2/1.0 (49 yrs)	Tubular visual field with temporal islands	non-recordable	Pale optic disc, narrow vessels and bone spicule pigmentation in mid-periphery	NCD	NCD	NCD
RP-0043: II:2	c.1485 + 2T>C/ c.1485 + 2T>C	Color disturbances and field constriction (32 yrs)	0.12/1.0	Relative central scotoma and some peripheral islands	NCD	Small optic disc, unstructured macula and atrophy in the periphery	Altered	NCD	Superficial punctate keratitis

ID, identification code; yrs, years; BCVA, best corrected visual acuity; OD, right eye; OS, left eye; ERG, electroretinogram; mf, multifocal; OCT, optical coherence tomography; NB, night blindness; VA visual acuity; RPE, retinal pigment epithelium; NCD, no clinical data.

**Table 2:** Homozygosity mapping results in families RP-0763 and RP-0043.

**Family**	**Ranking**	**Chr**	**Region of homozygosity**	**Region size**	**Number of genes**	**Known RD genes**
**RP-0763**	1	1	53.8–81.1	27.34	220	*RPE65*
	2	13	102.1–111.7	9.53	41	
	3	10	85.7–91.3	5.71	78	*CDHR1, RGR*
	4	9	65.3–70.8	5.5	29	
	5	17	72.5–74.8	2.34	48	
**RP-0043**	1	10	72–118.9	46.9	401	*CDHR1*
	2	4	144.5–162.2	17.7	83	*LRAT*
	3	10	14–27.8	13.8	78	
	4	19	36–38.5	2.5	84	*AIPL1*
	5	3	95.6–97.4	1.8	2	
	6	22	43.9–45.2	1.3	17	
	7	4	183.7–184.7	1	11	

Chr, Chromosome; RD, Retinal degeneration. Regions of homozygosity and regions’ sizes are provided based on the human reference sequence (hg19) and are expressed in megabases (Mb).

**Table 3:** *In silico* analysis of *CDHR1* intron 13 donor splice site potential as predicted by NetGene 2 Server and the Human Splicing Finder (HSF) analysis tools.

**Genomic location**	**Prediction software**	**Wt sequence**	**Mutant sequence**	**Reference score**	**Mutation score**	**Variation (%)**
Chr10: 85,970,922–85,970,923	HSF	aca**gt**ggg	aca**g**cggg	2.99	−4.75	−285.86
	NetGene2			0.273	0.043	15.75

The nucleotide position of the donor splice site is provided based on the human reference sequence (hg19). The two invariant nucleotides of the canonical 5′ splice site are bolded in the wild-type (wt) sequence. The mutant nucleotide is underlined in the box of the mutant sequence. The reference score refers to the wt sequence of the donor splice site. The Mutations Scores refers to the mutant sequence of the donor splice site. The Variation index measures the ratio of the mutation score divided by the reference score, expressed as a percentage.
