# Supplementary material for: Identification of two novel mutations in CDHR1 in consanguineous Spanish families with autosomal recessive retinal dystrophy
Source: Sci Rep. 2015 Sep 9;5:13902. doi: 10.1038/srep13902 (PMC4642573; doi:10.1038/srep13902)

SUPPLEMENTARY INFORMATION

Identification of two novel mutations in *CDHR1* in consanguineous Spanish families with autosomal recessive retinal dystrophy

Konstantinos Nikopoulos[#], Almudena Avila-Fernandez[#], Marta Corton, Maria Isabel Lopez-Molina, Raquel Perez-Carro, Lara Bontadelli, Silvio Alessandro Di Gioia, Olga Zurita, Blanca Garcia-Sandoval, Carlo Rivolta[#], Carmen Ayuso[#]*.

# Equally contributing authors

* Corresponding author

SUPPLEMENTARY INFORMATION INDEX

LEGEND TO SUPPLEMENTARY FIGURE

Supplementary Figure S1. Visual field testing of affected individual II:2 from family RP-0763. **(a)**: Visual field testing for both left and right eye performed at the age of 42 years showing tubular visual field with a small island of central and temporal vision. **(b)**: Visual field testing for both and left eye performed at the age of 45 years showing complete scotoma.

Supplementary Figure S1. Visual field testing of affected individual II:2 from family RP-0763. (a): Visual field testing for both left and right eye performed at the age of 42 years showing tubular visual field with a small island of central and temporal vision. (b): Visual field testing for both and left eye performed at the age of 45 years showing complete scotoma.

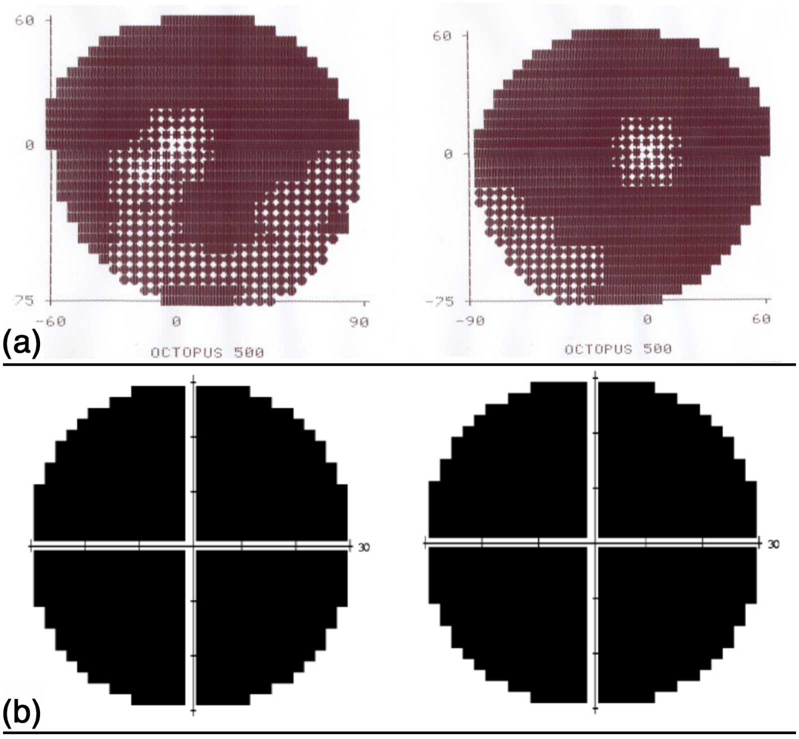

Supplement: Supplementary Information [file srep13902-s1.pdf]
